# A deletion variant Arg616 of androgen receptor in a Chinese family with complete androgen insensitivity syndrome

**DOI:** 10.3389/fgene.2023.1140083

**Published:** 2023-05-09

**Authors:** Leilei Ding, Duoduo Zhang, Fengxia Yao, Min Luo, Shan Deng, Qinjie Tian

**Affiliations:** ^1^ National Clinical Research Center for Obstetric and Gynecologic Diseases, Department of Obstetrics and Gynecology, Peking Union Medical College Hospital, Peking Union Medical College, Chinese Academy of Medical Sciences, Beijing, China; ^2^ Clinical Research Laboratory, Peking Union Medical College Hospital, Peking Union Medical College, Chinese Academy of Medical Sciences, Beijing, China; ^3^ Center for Rare Diseases Research, Chinese Academy of Medical Sciences, Beijing, China

**Keywords:** androgen receptor, complete androgen insensitivity syndrome, gonadal malignancy, variant, X-linked recessive inheritance

## Abstract

**Background:** Complete androgen insensitivity syndrome (CAIS, OMIM; 300068) is a disorder of sex development with X-linked recessive inheritance. Cases of CAIS usually present as female phenotype, with primary amenorrhea and/or inguinal hernia. Family aggregation is a rare scenario.

**Methods:** This study is a retrospective analysis of CAIS cases in a three-generation pedigree. The patients’ genomes were determined by sequencing the *androgen receptor* (*AR*) gene. The clinical data of the patients, including manifestations, hormone levels, and *AR* variants, were analyzed.

**Results:** Sixteen people in this family were involved. A deletion variant (c.1847_1849del; p. Arg616del) was identified in exon 3 of *AR,* which encodes the DNA binding domain. Until now, four patients and four carriers have been identified in three generations of this family. All the patients live as female, and one has developed gonadal malignancy.

**Conclusion:** The present study identified a deletion variant in three generations of a family with CAIS, including four carriers and four patients. This study verified the genetic pattern and the corresponding clinical characteristics of CAIS. Furthermore, a case with gonadal malignancy was discovered. The information on diagnosis and treatment in this pedigree is useful for prenatal diagnosis and genetic counseling of similar families.

## 1 Introduction

Complete androgen insensitivity syndrome (CAIS) is a rare X-linked recessive hereditary disorder, which affects the sexual development of XY embryos through complete androgen resistance (Batista et al., 2018). CAIS involves mutations in the Xq11-q12 region of the *androgen receptor* (*AR*) gene (Radpour et al., 2013). The effect of androgens depends mainly on their direct interactions with AR encoded by the *AR* gene (Verhoeven and Swinnen, 1999), which contains eight exons and encodes 920 amino acid residues (Chamberlain et al., 1996; Sack et al., 2001). AR is a single-strand polypeptide composed of four functional domains: the N-terminal domain (NTD), the DNA-binding domain (DBD), the hinge domain, and the C-terminal ligand-binding domain (LBD) (Tsai and O'Malley, 1994). *AR* mutations are identified in >95% of patients with CAIS, 70% of which are maternally inherited mutations; the remaining 30% are *de novo* mutations (Oakes et al., 2008; Zhang et al., 2021).

Androgens are responsible for the proper development of internal and external genitalia during the fetal stage (Alemany, 2022) and promote male secondary sexual characteristics in adolescence and stimulate muscle and bone growth, spermatogenesis, and fertility in adults (Yamada et al., 2006). The most common presentation of CAIS is the occurrence of unilateral or bilateral inguinal hernias in infants or children; however, some individuals are not diagnosed until adolescence due to primary amenorrhea.

In this study, we report a variant in the DBD region of *AR* across three generations from a single Chinese pedigree with CAIS. A hemizygous three-base deletion was identified in exon 3 of *AR* (GenBank NM_000044.6: c.1847_1849del), leading to the deletion of Arg616. This study also discusses the genetic variant, hereditary pattern, and genetic consultation in this family.

## 2 Patients and methods

### 2.1 Family and patients

**FIGURE 1:**
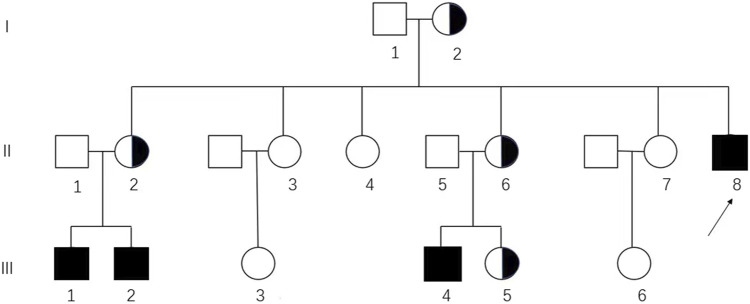
Pedigree analysis of the Chinese family affected by CAIS. The genotypic 46, XX and 46, XY individuals are represented by circles and squares, respectively. Black squares, affected individuals; semi-black circles, carriers; arrows, proband (II-8) in this pedigree.

The clinical data of this CAIS pedigree were retrospectively collected from medical records and telephone inquiries (Figure 1). The study was approved by the Ethics Committee of Peking Union Medical College Hospital (PUMCH) (IRB Number: JS-2510) and written consent to participate and consent to publication were obtained from each individual or legal guardian if underage. The data used in this study were anonymous or kept confidential. This study did not affect the diagnosis or treatment of the patients.

### 2.2 Clinical data

Clinical information was summarized, including the chief complaints and signs related to sexual development, serum hormone levels, surgical information, and pathological results. We used the Tanner stage to describe the development of breasts or pubic or axillary hair. Patient height, weight, and body mass index (BMI) were documented at the first visit. The hormone levels, including serum follicle-stimulating hormone (FSH), luteinizing hormone (LH), testosterone (T), estradiol (E2), and progesterone (P), were measured on an automated Elecsys Immunoanalyzer (Beckmann, United States) system.

### 2.3 AR analysis

Targeted Sanger sequencing of *AR* was performed to evaluate the variant. Peripheral blood samples from family members including the proband (II-8), her sisters (II-2, II-3, II-4, II-6, and II-7), and her parents (I-1 and I-2) were sent for *AR* testing. Additionally, blood samples from the husbands and daughters of her sisters (II-1, II-5, III-1, III-2, III-3, III-4, III-5, and III-6) were also tested for *AR* (Figure 1).

The Lab-Aid 820 automatic DNA extraction kit (Xiamen Zhishan Biotechnology Co., Ltd., China) was used to extract genomic DNA from the peripheral blood samples. The eight exons and intron-exon boundaries of *AR* (GenBank accession number NM_000044) were amplified by polymerase chain reaction (PCR) with primers designed by Primer3 online software (Table 1). The PCR volume was 25 μL, containing 20–100 ng genomic DNA, 12.5 μL PCR master mix (Beijing Tianyihuiyuan Biological Company), and 10 μM of each primer. The regions of candidate SNVs were amplified according to the manufacturer’s instructions.

**TABLE 1:** Primers for *AR* amplification and sequencing.

Exon	Primers	Sequence (5′-3′)	Primer length (bp)	Fragment length (bp)
1	ARe1-1	F: GAC​TAC​CGC​ATC​ATC​ACA​GC	20	495
		R: CTG​GGA​CGC​AAC​CTC​TCT​C	20	
1	ARe1-2	F: CAG​CAA​GAG​ACT​AGC​CCC​AG	20	585
		R: CAA​AAG​TGG​GGC​GTA​CAT​GC	19	
1	ARe1-3	F: TCG​ACC​ATT​TCT​GAC​AAC​GC	20	551
		R: CTCGCCAGGTCCCCATAG	18	
1	ARe1-4	F: CAA​CTT​TCC​ACT​GGC​TCT​GG	20	649
		R: TTT​ACC​CTG​CTG​AGC​TCT​CC	20	
1	ARe1-5	F: CCCCTACGGCTACACTCG	18	281
		R: CAA​TCT​GAG​TGT​TCG​CGC​AG	20	
2	Are2	F: GCT​TCA​CAC​TAA​CTA​ACT​TGA​GC	23	373
		R: AAA​ATC​CTG​GGC​CCT​GAA​AG	20	
3	Are3	F: GGT​GCC​ATA​CTC​TGT​CCA​CT	20	383
		R: GTC​AGC​CTG​TGT​CTA​GAG​CA	20	
4	Are4	F: GTG​TTG​AAT​GAG​CAC​TTG​TCC​T	22	582
		R: AAC​AAT​CCC​TCT​CCC​ACA​GG	20	
5	Are5	F: GGA​TGC​CCG​AAT​ACC​AGA​G	19	394
		R: TCA​TAC​TGG​ATT​GGC​TGG​CT	20	
6	Are6	F: GCA​GGA​GAA​ACA​GCA​AGC​T	20	351
		R: AGG​AGC​TGG​CTT​TTC​CCT​AA	20	
7	Are7	F: GGG​GTC​AAG​TCT​GTG​GTC​A	19	389
		R: TCT​TCC​TGG​ACC​ACA​CTC​AA	20	
8	Are8	F: GGG​GAG​GAA​ACA​AAA​GGC​T	20	487
		R: GTG​CCA​TGG​GAG​GGT​TAG​AT	20	

Abbreviations: F, forward; R, reverse.

For Sanger sequencing, the extracted genomic DNA was sequenced with the BigDye Terminator Cycle Sequencing Ready Reaction Kit, version 3.1 (Applied Biosystems; Thermo Fisher Scientific, Inc., Waltham, MA, United States) according to the manufacturer’s instructions. The sequences were analyzed on an ABI 3130 Genetic Analyzer (http://tools.thermofisher.com/content/sfs/manuals/4477796.pdf). The sequencing results were compared with the reference genomic sequence obtained from the UCSC Genome Browser (Santa Cruz, CA, United States; https://genome.ucsc.edu/). Variants were confirmed based on the Human Gene Mutation Database (HGMD) and the NCBI dbSNP database (http://www.ncbi.nlm.nih.gov/SNP).

## 3 Results

### 3.1 Clinical manifestations and hormone profiles

Four patients were identified in this family. Their chromosomal analysis confirmed a 46, XY karyotype. II-8 was the proband in this pedigree. The clinical manifestations and hormone profiles of the four patients (II-8, III-2, III-4, and III-1) are presented in Table 2.

**TABLE 2:** Patient clinical characteristics.

Clinical characteristic	II-8	III-2	III-4	III-1
Age of presentation (years)	15	16	7	2
Chief complaint	Inguinal hernia and primary amenorrhea	Primary amenorrhea	Inguinal hernia	Inguinal hernia
Height (cm)	167	167	127	96
BMI (kg/m^2^)	17.57	22.94	15.50	16.62
Breast	IV	IV	I	I
Menstruation	No	No	No	No
Juvenile female external genitalia	Yes	Yes	Yes	Yes
Blind-ended vagina	6 cm	2 cm	6 cm	NA
Cervix	No	No	No	NA
Pubic/axillary hairs	I	I	I	I
Masses	Masses in the pelvis and abdomen, left inguinal hernia	No masses in both inguinal regions	A 1.5 cm mass in the right inguinal region	A 3 cm mass in the right inguinal region
FSH (IU/l)	86.9	4.67	NA	7.10
LH (IU/l)	112.04	24.31	NA	0.72
P (ng/ml)	0.86	1.49	0.05	0.089
T (ng/ml)	1.82	8	0.2	0.12
E2 (pg/ml)	21	49	<20	1.20
B-scan uterus or bilateral ovaries gonads	No uterus or bilateral ovaries	No uterus or bilateral ovaries	No uterus or bilateral ovaries	No uterus or bilateral ovaries
	Large pelvic mass, malignant lesions considered		Cryptorchidism in the right inguinal	Cryptorchidism in the right inguinal
	Left inguinal hernia	Gonads in pelvis	region and left lower abdominal cavity	region and left lower abdominal cavity
Surgery	Bilateral gonadectomy + tumor cytoreduction	Laparoscopic bilateral gonadectomy	Not yet	Not yet
Post-operative histopathology	Yolk sac tumor + seminoma (right gonad) and testis (left gonad)	Under-developed testes	NA	NA

The reference ranges for female sex hormones during the follicular stage are as follows: LH, 2.12–10.89 mIU/ml; FSH, <10 mIU/ml; T, 0.10–0.84 ng/ml; E2, 22–115 pg/ml; and P, 0.38–2.28 ng/ml. NA: not available.

The proband (II-8, 26 years) was the first from this pedigree to visit our hospital after 11 years of inguinal hernia and primary amenorrhea. Gonadal malignancy was identified during further treatment. Her niece (III-2, 16 years) developed the same symptom of primary amenorrhea. The ultrasound at the local hospital showed a congenital absence of a uterus. For further treatment, she was admitted to undergo an operation. After their diagnosis of CAIS, her 8-year-old younger niece (III-4) presented to outpatient clinics with findings of inguinal masses for 1 year. Additionally, a 3 cm mass protruding from the right groin after walking or running was observed in her 2-year-old niece (III-1). She was diagnosed genetically as a patient of CAIS without any secondary sexual developmental abnormalities.

Except for the 2-year-old girl without physical examination, the other three patients all presented a blind-ended vagina and lack of cervix. The absence of a uterus was confirmed by ultrasound in the four patients. Regarding secondary sexual characteristics, neither of the two postpubertal patients (II-8 and III-2) showed well-developed pubic or axillary hair, and Tanner stage IV breast development that was consistent with a typical CAIS, while the prepubertal patients (III-4 and III-1) were consistent with their age.

Preoperative sex hormone testing showed serum levels of E2 and P in II-8 and III-2 in agreement with the lower early follicular phase. The T levels were elevated to the normal male range or significantly higher than the female average. The gonadotropin level was normal in III-2 but was increased significantly to the postmenopausal level in II-8. III-4 and III-1 presented with normal sex hormone levels consistent with their age.

The proband (II-8) developed a malignancy of the gonads and received cytoreductive surgery. Pathology confirmed a yolk sac tumor, seminoma, and testis. The postoperative pathological stage was IIIc according to the National Comprehensive Cancer Network guidelines. After surgery and four courses of chemotherapy with a combination of bleomycin, cisplatin, and etoposide, the cancer antigen 125 (CA125) level plummeted from 455 U/ml to 6.5 U/ml, and the alpha-fetoprotein (AFP) level from 804 ng/ml to 2 ng/ml. Proper hormone replacement therapy was administered. III-2 had received laparoscopic bilateral gonadectomy, in which the histological analysis demonstrated underdeveloped testes of both gonads.

### 3.2 Genetic analysis of *AR*

In this family, four hemizygous mutations (II-8, III-1, III-2, and III-4) and four heterozygous mutations (I-2, II-2, II-6, and III-5) were identified (Supplementary Figure S1). Pedigree analysis showed that the variant was from matrilineal inheritance (Figure 1).

**FIGURE 2:**
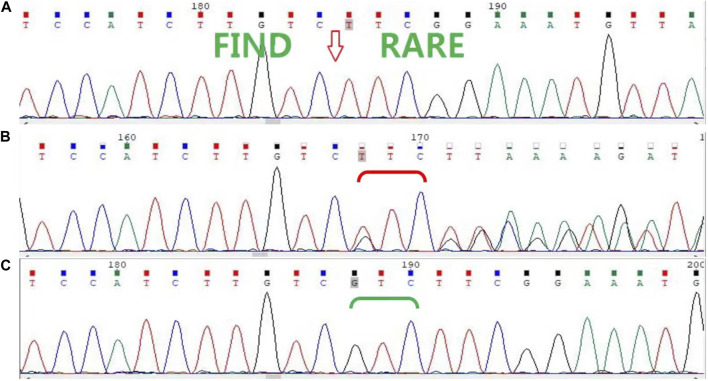
Sanger sequencing of *AR* in the family. **(A)** Sanger sequencing confirmed that the proband (II-8) and her nieces (III-1, III-2, and III-4) have the c.1847_1849del (p. Arg616del) variant. **(B)** The proband’s mother (I-2), sisters (II-2 and II-6), and another niece (III-5) are heterozygous carriers, **(C)** while her father (I-1) and other sisters (II-3, II-4, and II-7) are wild type.

In this family, a deletion variant (c.1847_1849del; p. Arg616del) was identified in exon 3 of *AR,* which encodes the DBD, and has been previously reported (Figure 2). The c.1847_1849del (p. Arg616del) is a known variant included in the HGMD database and had been detected in an androgen-insensitive pedigree (PMID: 8162033). This variant causes a change in protein length (PM4). This variant was not detected in the Genome Aggregation Database (gnomAD) normal population database (PM2_Supporting). Based on available evidence, according to the 2015 American College of Medical Genetics and Genomics (ACMG) guideline, this variant was defined as a variant of unknown clinical significance (PM4+PM2_Supporting). Although this variant was defined as a variant of unknown clinical significance, it caused clinical symptoms in our study, leading to the occurrence of CAIS in this family.

## 4 Discussion

This study describes three generations of a family with CAIS, including 16 people and four patients. The chief complaints to the medical service were groin mass or primary amenorrhea. The four patients in this family were raised as female due to the lack of male phenotype. Since three members of this family visited the hospital successively and there was a phenomenon of family aggregation, the whole *AR* gene of the family members was sequenced to explore the pathogenesis. Sanger sequencing showed an *AR* deletion variant c.1847_1849del in this family, which was first reported in an androgen-insensitive family in 1994 (Beitel et al., 1994). The X-linked recessive inheritance pattern of CAIS was well-validated in this family. As the carrier of the variant, I-2 passed it on to the next generation. This pedigree was valuable and it was rare for the grandparents (I-2) to have raised six daughters in the era of family planning policy in China in the 1970s. Clinical and genetic data from the whole pedigree were collected abundantly and thoroughly. Moreover, *AR* was sequenced simultaneously in all blood samples of 16 people of this pedigree.

Arg616 is a strictly conserved residue in the DBD in all members of the superfamily, such as steroid, thyroid, and vitamin D receptor proteins. Beitel et al. (1994) reported that Arg616 is an invariant residue in the C-terminal a-helix of the DBD. They predicted that Arg616 contributes to the normal tetrahedral arrangement of the four cysteines at the base of the C-terminal zinc finger, and to its associated α-helices. Therefore, the Arg616 deletion causes CAIS by perturbing the normal androgen receptor-androgen response element (AR-ARE) interaction. Our results showed that the Arg616 deletion mutation in the DBD region, exon 3, is the pathogenic cause of CAIS in this Chinese family. However, functional verification in cell and animal experiments is lacking, and the genotype-phenotype correlation requires further study.

Our genetic analysis revealed that the patients inherited the variant from their maternal line. The 46XY progeny carrying the mutated gene can develop CAIS. As an X-linked recessive genetic disease, the three-level prevention of CAIS is advocated. The primary prevention is carrier screening. It is important to identify heterozygous carriers of gene mutations for genetic counseling. *AR* testing is recommended for women with a family history of CAIS. In this study, we revealed the existence of the exon 3 p. Arg616del variant in this family. Therefore, it would be more economical to assess only exon 3 instead of sequencing the entire *AR* gene in future genetic tests in this family. The secondary prevention is prenatal diagnosis. The diagnosis can be confirmed or excluded by amniocentesis, villus sampling for fetal chromosome karyotype analysis, and genetic testing. The phenotype of external genital development can be determined by imaging examinations (ultrasound and magnetic resonance imaging) during pregnancy, to determine the consistency of fetal chromosomal sex and external genital phenotype. Tertiary prevention focuses on postnatal physical examinations. Unclear female vulva or genitalia sex or 46XY chromosome karyotype, combined with CAIS family history, can be considered for the diagnosis of CAIS (罗敏 and 姚凤霞,^2021^). Further genetic testing can be performed to determine the cause, providing the possibility of genetic counseling for future pregnancies.

II-8 had experienced gonadal malignancy (testicular germ cell tumor), which was first identified in patients with CAIS with this p. Arg616del variant. Chung et al. reported that gonadal malignant transformation was related to a variety of factors, including testicular location, individual genetic susceptibility, and residual androgen activity (Chung et al., 2013). Oncogenetic susceptibility may also be associated with one or more single nucleotide polymorphisms (SNPs) (Kratz et al., 2011). In this study, all patients carried the same variant and had ectopic gonads, but thus far only II-8 had developed gonadal malignancy. We speculated that the tumor was not necessarily associated with the variant; rather, it may be related to the gonad location, residual androgen activity, or the postponement of gonadectomy. The literature indicates that the risk of malignant progression increases with age (Cools et al., 2005; Cools et al., 2006). Therefore, testes excision is of great significance for this family of CAIS cases.

The timing of gonadectomy in patients with CAIS remains controversial. If prophylactic orchiectomy is performed, the occurrence of testicular germ cell tumor (TGCT) in patients with CAIS can be reduced. But the reservation of testicular tissue produces androgens and can be converted to estrogen by aromatase to activate pubertal development (Lanciotti et al., 2019). The reported incidence of TGCT is >22% in adulthood (Deans et al., 2012), while the incidence in childhood and adolescence is very low. In the Morris and Mahesh study of 181 cases of AIS, among patients with gonadal tumors, only one was a teenager and two were patients in their 20s (Hurt et al., 1989). Most malignancies in patients with CAIS after puberty are less invasive (Tack et al., 2018). Therefore, bilateral inguinal hernia in childhood is no longer an indication for early gonadectomy (Döhnert et al., 2017). Most patients who wish to live as female can postpone bilateral gonadectomy to post-puberty after pubertal breast development, followed by estrogen replacement therapy. However, early diagnosis and regular follow-up are still required. Ultrasound remains the first-line assessment method for gonads (Kim et al., 2007). From adolescence, annual ultrasound follow-up is recommended (Looijenga and Cools, 2017). Some classic serum markers (β-HCG, AFP, and LDH) and hormonal assessments (FSH, LH, T, and inhibin B) can also be used as a follow-up in patients with CAIS (Cools et al., 2017). Although current treatment advances advocate postponing gonadectomy until after puberty, in this family, due to the occurrence of gonadal malignancy, we advocate gonadectomy relatively earlier to prevent such malignancy.

## 5 Conclusion

An X-linked inheritance variant in *AR* was identified in a three-generation family involving 16 people. The variant c.1847_1849del (p. Arg616del) was highly conserved and was verified to perturb normal androgen–AR interactions. This variant severely disrupted androgen activity, with typical clinical characteristics of CAIS. Furthermore, a case with gonadal malignancy was diagnosed. These findings may be useful for the prenatal diagnosis and genetic counseling of the offspring of this pedigree or relevant families with CAIS.

## Data Availability

The datasets for this article are not publicly available due to concerns regarding participant/patient anonymity. Requests to access the datasets should be directed to the corresponding author.
