# Supplementary material for: A deletion variant Arg616 of androgen receptor in a Chinese family with complete androgen insensitivity syndrome
Source: Front Genet. 2023 May 9;14:1140083. doi: 10.3389/fgene.2023.1140083 (PMC10236311; doi:10.3389/fgene.2023.1140083)
Supplement: Supplementary file 1 [file DataSheet1.pdf]

Supplementary Material

A deletion variant Arg616 of androgen receptor in a Chinese family with complete androgen insensitivity syndrome

Leilei Ding[1†], Duoduo Zhang[1†], Fengxia Yao[3], Min Luo[1], Shan Deng[1], Qinjie Tian[1,2*]

*** Correspondence:** Qinjie Tian
E-mail: qinjietn@163.com

Supplementary Data（the CARE checklist ）

1. Title – The diagnosis or intervention of primary focus followed by the words “case report”.

A deletion variant Arg616 of androgen receptor in a Chinese family with complete androgen insensitivity syndrome

Title specifies a family study.

2. Key Words – 2 to 5 key words that identify diagnoses or interventions in this case report (including "case report").

NA: The style of our manuscript is original article.

androgen receptor (AR), complete androgen insensitivity syndrome (CAIS), gonadal malignancy, variant, X-linked recessive inheritance

3. Abstract – (structured or unstructured)

structured

Introduction – What is unique about this case and what does it add to the scientific literature?

Cases of CAIS usually presented as female phenotype, primary amenorrhea, and/or inguinal hernia. Family aggregation is a rare scenario. Sixteen people in this family were involved, a deletion variant (c.1847_1849del; p. Arg616del) was identified in exon3 of the AR gene that encodes the DNA binding domain.

The patient’s main concerns and important clinical findings.

Until now, four patients and four carriers have been found in three generations of this family. All the patients live as female and one of them has been found to have gonadal malignancy and received cytoreductive surgery.

The primary diagnoses, interventions, and outcomes.

NA. Because the cases involved in this article were all genetically diagnosed, they could be diagnosed in time.

Conclusion – What are one or more “take-away” lessons from this case report?

The present study identified a deletion variant in a CAIS family of three generations that involved four carriers and four patients, verified the genetic pattern and the corresponding clinical characteristics of CAIS. Furthermore, a case with gonadal malignancy was discovered. The information on diagnosis and treatment in this pedigree is useful for the prenatal diagnosis and genetic counseling of a similar family.

4. Introduction

– Briefly summarizes why this case is unique and may include medical literature references.

The most common presentation of CAIS is the occurrence of unilateral or bilateral inguinal hernias in infants or children; however, there are some individuals who are not diagnosed until teenage due to primary amenorrhea.

In this study, we report a variant in the DBD region of the AR across three generations involved 16 people from a single Chinese pedigree with CAIS. It is not easy to collect such comprehensive family information and make genetic diagnosis. A hemizygous deletion of 3 bases was identified in the AR (GenBank NM_000044.6: c.1847_1849del) in exon 3, leading to a deleterious deletion of Arg616. The genetic variant, hereditary pattern, and genetic consultation in this big family were discussed in this paper. we conducted gene sequencing analysis on almost every member, which confirmed the genetic pattern of CAIS and the pathogenicity of this mutation site, and enriched the mutation Database of AR genes.

5. Patient Information

De-identified patient specific information.

Clinical information was summarized, including the chief complaint and signs related to sexual development, serum hormone as well as surgical information and pathological results. We use the Tanner stage to describe the development of breast, pubic or axillary hair. Patients’ height, weight and body mass index (BMI) were documented at the first visit. The levels of the hormones were measured by the automated Elecsys Immunoanalyzer (Beckmann, USA), including serum follicle-stimulating hormone (FSH), luteinizing hormone (LH), testosterone (T), estradiol (E2), and progesterone (P).

Primary concerns and symptoms of the patient.

groin mass or primary amenorrhea

Medical, family, and psychosocial history including relevant genetic information.

In this family, four hemizygous mutations (II-8, III-1, III-2 and III-4) and four heterozygous mutations (I-2, II-2, II-6 and III-5) were found. Pedigree analysis showed that the variant was from matrilineal inheritance.

Relevant past interventions and their outcomes.

NA.

6.Clinical Findings

– Describe significant physical examination (PE) and important clinical findings.

The proband (II-8, 26 years) was the very first to visit our hospital after 11 years of inguinal hernia and primary amenorrhea from this pedigree. Gonadal malignancy was found during further treatment. Her niece (III-2,16 years) developed the same symptom of primary amenorrhea and the ultrasound of the local hospital showed congenital absence of uterus. For further treatment, she was admitted for operation. After their diagnosis of CAIS, her 8-year-old younger niece (III-4) presented to out-patient clinics with finding of inguinal masses for one year. What's more, there was a 3cm mass protruding from the right groin after walking or running in her 2-year-old niece (III-1). She was diagnosed genetically as a patient of CAIS without any secondary sexual developmental abnormalities.

Except for this 2-year-old infantile girl without physical examination, the other three patients all presented a blind-ended vagina and loss of cervix. The absence of uterus confirmed by ultrasound in the four patients. In terms of the secondary sexual characteristics, the two postpubertal patients (II-8 and III-2) neither showed well-developed pubic or axillary hair, and Tanner stage IV breast development that was consistent with a typical CAIS, while the prepubertal patients (III-4 and III-1) are consistent with their age.

7. Timeline – Historical and current information from this episode of care organized as a timeline (figure or table).

NA.

8. Diagnostic Assessment

Diagnostic methods (PE, laboratory testing, imaging, surveys).

Diagnostic challenges.

Diagnosis (including other diagnoses considered).

Prognostic characteristics when applicable.

CAIS is the most common type of AIS. Only a small number of patients can be diagnosed before or soon after birth due to the paradox between the 46, XY karyotype from prenatal diagnosis and the female phenotype discovered at birth. However, more common presentation is the occurrence of bilateral inguinal hernias in infants or children, usually bilateral, and there are still some individuals who are not diagnosed until teenage due to primary amenorrhea. They have no uterus, no menstrual formation.

Initial diagnosis of the disease can be made by assessing clinical symptoms, hormone levels, chromosomal karyotypes, and gynecological ultrasound results. Combined with genetic testing results, patients and carriers can be diagnosed in a timely manner, enabling close follow-up and early treatment.

9. Therapeutic Intervention

Types of therapeutic intervention (pharmacologic, surgical, preventive).

Administration of therapeutic intervention (dosage, strength, duration).

Changes in therapeutic interventions with explanations.

The testis may be located in the abdominal cavity, the inguinal canal, or the labia major. Undescended testis may develop germ cell tumors, but the risk of tumors is considered low and early prophylactic gonadectomy is not recommended. However, the risk of tumor formation increases with age, so patients should be followed closely and gonadectomy should be performed after puberty if appropriate. Most patients who wish to live as female can postpone to post-puberty to have bilateral gonadectomy after pubertal breast development and followed by estrogen replacement therapy.

In this family, except for preadolescent III-4 and III-1, gonadectomy was performed in the remaining 2 adult patients. It should be noted that the proband (II-8) found malignancy of the gonads and received cytoreductive surgery.

10. Follow-up and Outcomes

Clinician- and patient-assessed outcomes if available.

Important follow-up diagnostic and other test results.

Intervention adherence and tolerability. (How was this assessed?)

Adverse and unanticipated events.

Undescended testis may develop germ cell tumors, so early diagnosis and regular follow-up are still necessary. Ultrasound remains the first-line assessment method for gonad. From adolescence, annual ultrasound follow-up is recommended. Some classic serum markers (such as β-HCG, AFP, LDH) and hormonal assessments (FSH, LH, T, and inhibin B) can also be used as follow-up in CAIS patients. Although current treatment advances advocate postponing gonadectomy after puberty, for this family, due to the occurrence of gonadal malignancy, we can advocate gonadectomy relatively earlier to prevent malignancy.

11. Discussion

Strengths and limitations in your approach to this case.

Discussion of the relevant medical literature.

The rationale for your conclusions.

The primary “take-away” lessons from this case report (without references) in a one paragraph conclusion.

Please see main manuscript-the part of discussion.

12. Patient Perspective – The patient should share their perspective on the treatment(s) they received.

NA.

13. Informed Consent – The patient should give informed consent. (If not, explain)

Our study was approved by the Ethics Committee of Peking Union Medical College Hospital (PUMCH) (IRB Number: JS-2510) and written consent to participate and consent to publication were obtained from each individual or legal guardian if underage. The data used in this study were anonymous or kept confidential. This study did not influence the diagnosis or treatment of the patients.

Supplementary Figures

Supplementary Figure 1. Sanger sequencing of the *AR* gene in the family.

III-2 carried the AR gene c.1847_1849del (p. Arg616del) mutation.

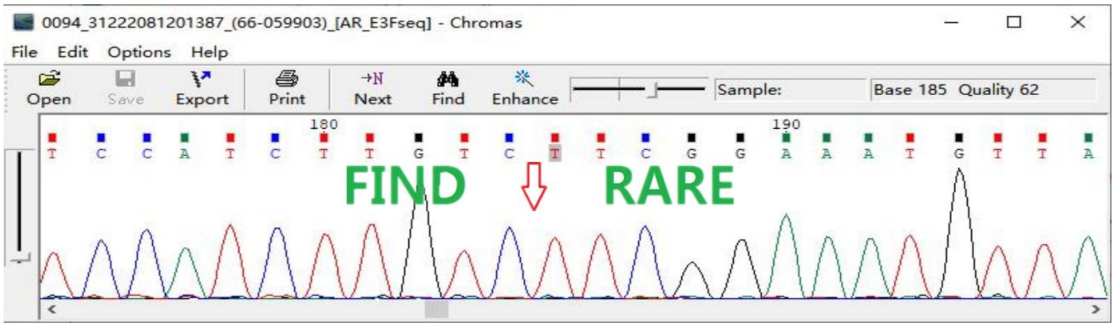

II-1 did not carry the AR gene c.1847_1849del (p.Arg616del) mutation.

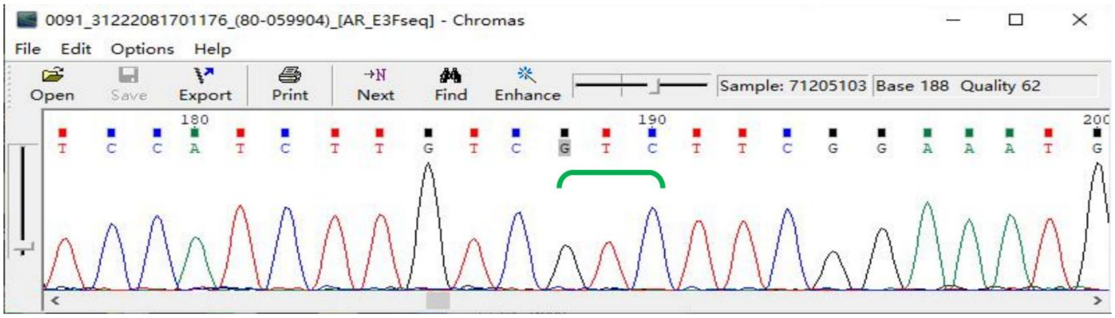

I-1 did not carry the AR gene c.1847_1849del (p.Arg616del) mutation.

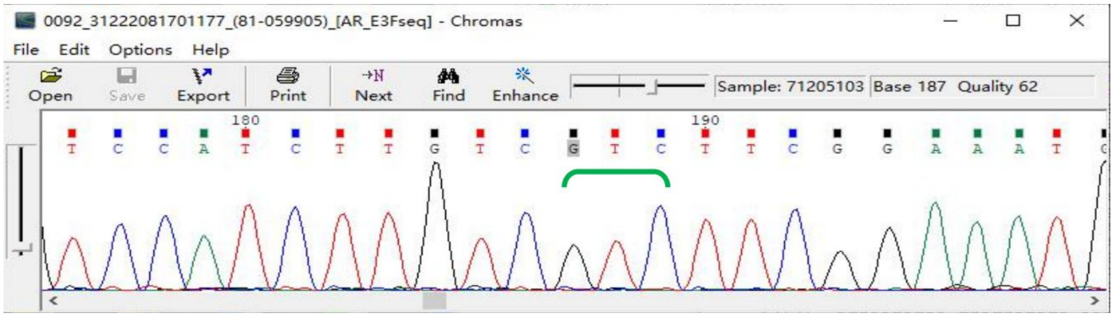

III-4 did not carry the AR gene c.1847_1849del (p.Arg616del) mutation..

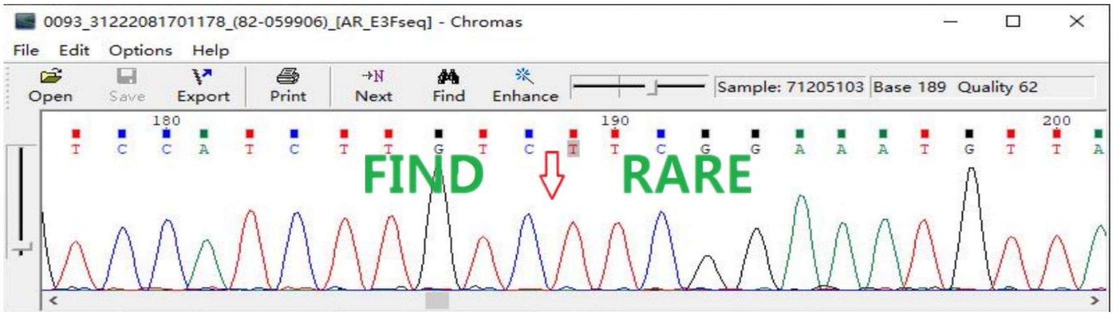

II-6 carried a heterozygous mutation of AR gene c.1847_1849del (p.rg616del).

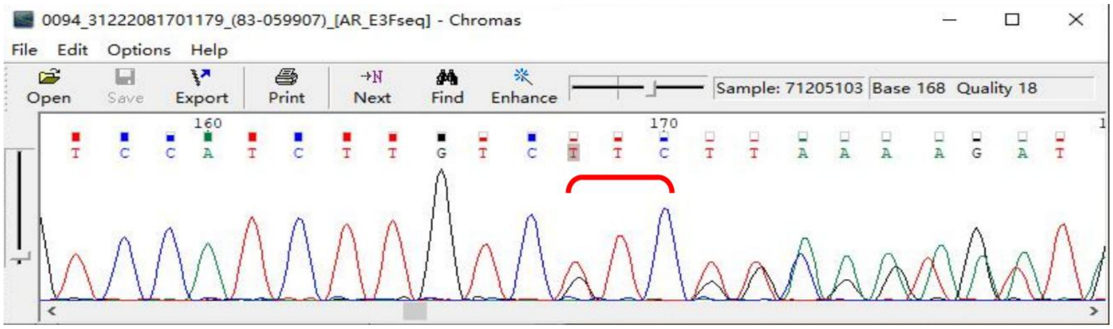

III-1 carried the AR gene c.1847_1849del (p.Arg616del) mutation..

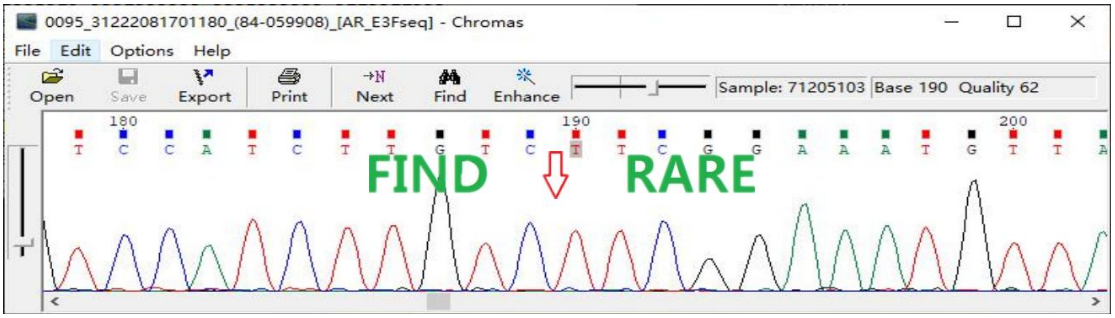

II-8 carried the AR gene c.1847_1849del (p.Arg616del) mutation..

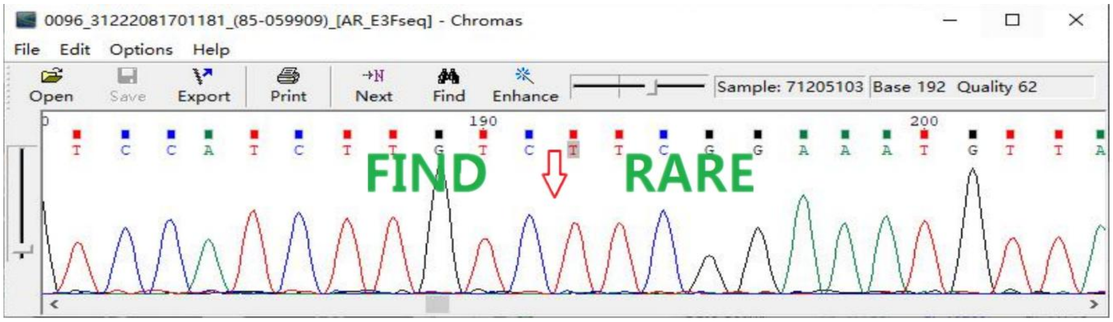

II-2 carried a heterozygous mutation of AR gene c.1847_1849del (p.rg616del).

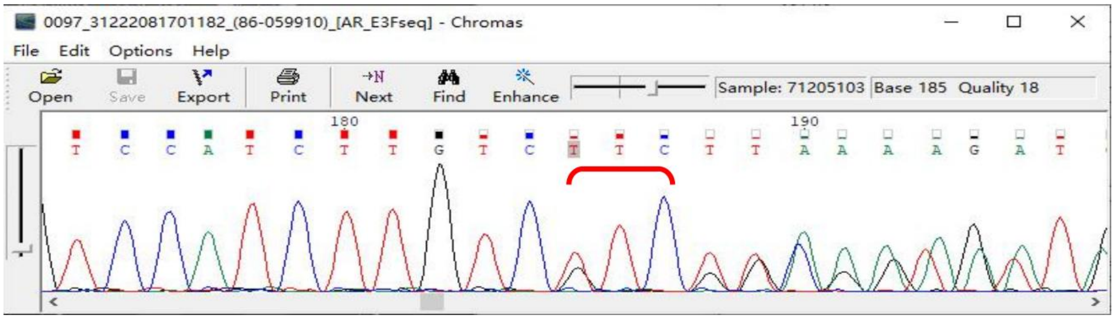

I-2 carried a heterozygous mutation of AR gene c.1847_1849del (p.rg616del).

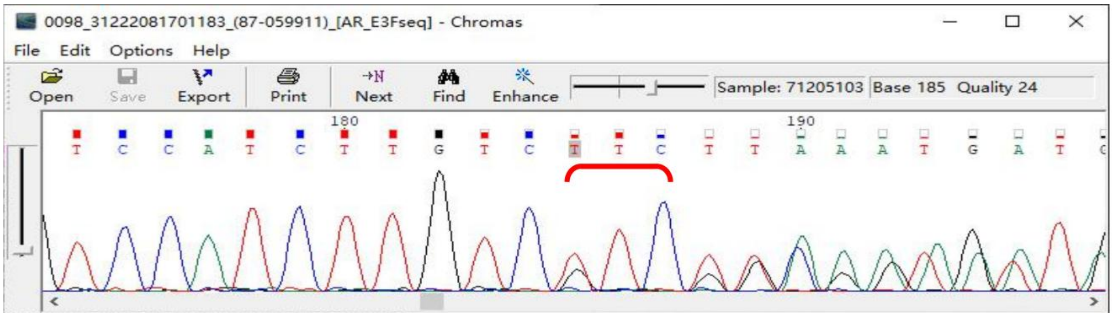

II-4 did not carry the AR gene c.1847_1849del (p.Arg616del) mutation..

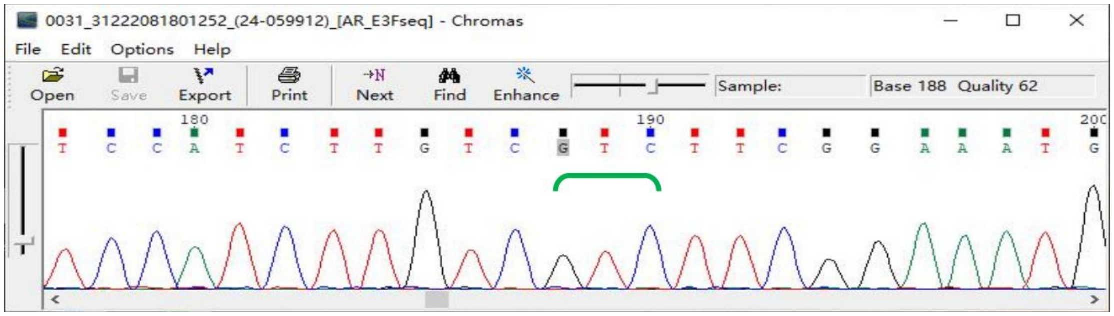

II-5 did not carry the AR gene c.1847_1849del (p.Arg616del) mutation..

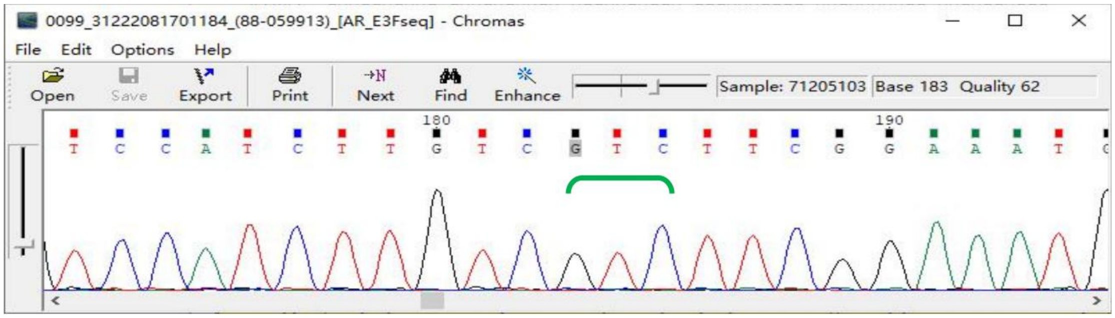

III-5 carried a heterozygous mutation of AR gene c.1847_1849del (p.rg616del).

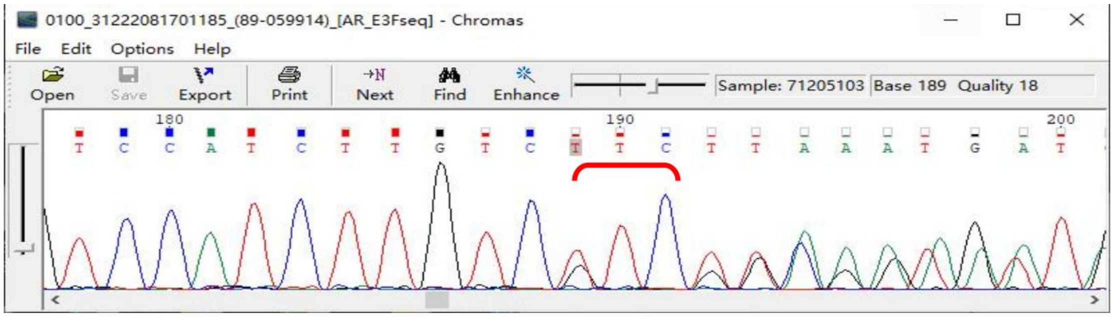

II-3 did not carry the AR gene c.1847_1849del (p.Arg616del) mutation..

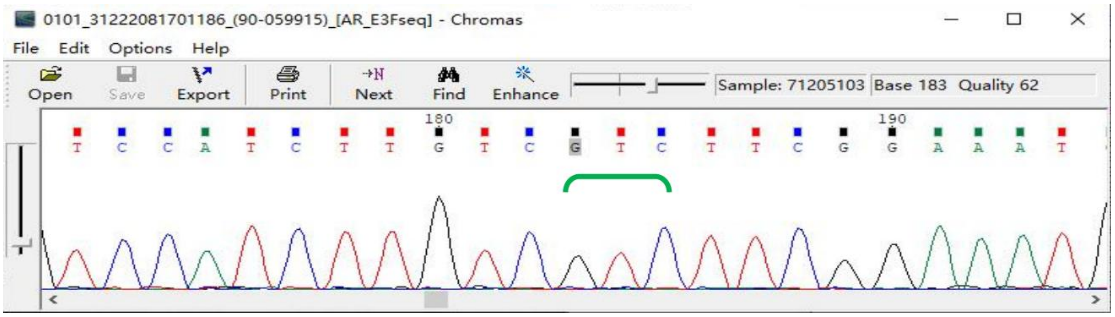

III-3 did not carry the AR gene c.1847_1849del (p.Arg616del) mutation..

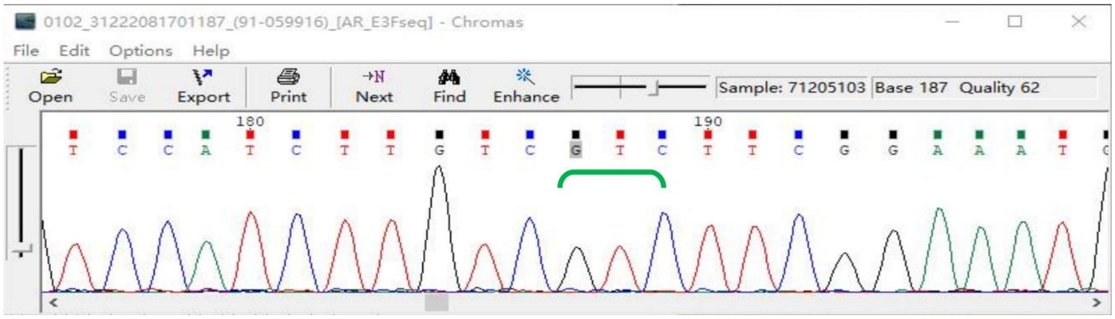

III-6 did not carry the AR gene c.1847_1849del (p.Arg616del) mutation..

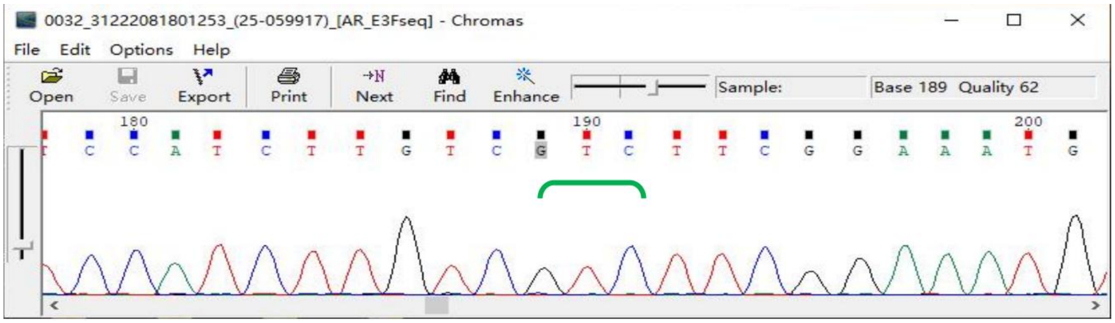

II-7 did not carry the *AR* gene c.1847_1849del (p.Arg616del) mutation..

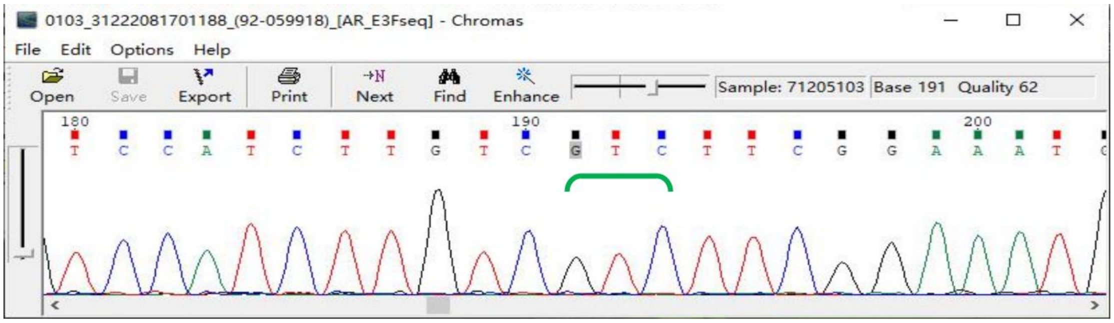
0103_31222081701188_(92-059918)_[AR_E3Fseq] - Chromas
File Edit Options Help
Open Save Export Print Next Find Enhance
Sample: 71205103 Base 191 Quality 62
180
190
200
T C C A T C T T G T C G T C T T C G G A A A T
